# Polyanhydride Nanovaccine Induces Robust Pulmonary B and T Cell Immunity and Confers Protection Against Homologous and Heterologous Influenza A Virus Infections

**DOI:** 10.3389/fimmu.2018.01953

**Published:** 2018-08-28

**Authors:** Zeb R. Zacharias, Kathleen A. Ross, Emma E. Hornick, Jonathan T. Goodman, Balaji Narasimhan, Thomas J. Waldschmidt, Kevin L. Legge

**Affiliations:** ^1^Interdisciplinary Immunology Graduate Program, Department of Pathology, University of Iowa, Iowa City, IA, United States; ^2^Department of Chemical and Biological Engineering and Nanovaccine Institute, Iowa State University, Ames, IA, United States; ^3^Department of Microbiology and Immunology, University of Iowa, Iowa City, IA, United States; ^4^Nanovaccine Institute, University of Iowa, Iowa City, IA, United States

**Keywords:** influenza virus, nanovaccine, adaptive immunity, tissue-resident memory, heterosubtypic protection

## Abstract

Influenza A virus (IAV) is a major cause of respiratory illness. Given the disease severity, associated economic costs, and recent appearance of novel IAV strains, there is a renewed interest in developing novel and efficacious “universal” IAV vaccination strategies. Recent studies have highlighted that immunizations capable of generating local (i.e., nasal mucosa and lung) tissue-resident memory T and B cells in addition to systemic immunity offer the greatest protection against future IAV encounters. Current IAV vaccines are designed to largely stimulate IAV-specific antibodies, but do not generate the lung-resident memory T and B cells induced during IAV infections. Herein, we report on an intranasally administered biocompatible polyanhydride nanoparticle-based IAV vaccine (IAV-nanovax) capable of providing protection against subsequent homologous and heterologous IAV infections in both inbred and outbred populations. Our findings also demonstrate that vaccination with IAV-nanovax promotes the induction of germinal center B cells within the lungs, both systemic and lung local IAV-specific antibodies, and IAV-specific lung-resident memory CD4 and CD8 T cells. Altogether our findings show that an intranasally administered nanovaccine can induce immunity within the lungs, similar to what occurs during IAV infections, and thus could prove useful as a strategy for providing “universal” protection against IAV.

## Introduction

Influenza A virus (IAV) is a common respiratory pathogen that undergoes seasonal antigenic drift continually giving rise to variant strains that can escape existing immune protection. This viral drift detrimentally impacts public health as well as the economy within the United States, which is exemplified by the ~310,000 hospitalizations, 12,000 deaths, and an $87 million-dollar financial burden observed during the 2015–2016 season (1, 2). Traditionally, the spread of IAV has been prevented by two vaccination strategies: inactivated influenza vaccine (IIV) and live-attenuated influenza vaccine (LAIV). Both IIV and LAIV primarily provide systemic immunity by inducing IAV-specific antibody responses (3, 4). However, it is less clear if these vaccination strategies generate robust *de novo* IAV-specific CD4 or CD8 T cell responses within the lower lung mucosa (4–7). Due to its intramuscular delivery, IIV is not thought to drive airway-resident effector T cell responses (6). Although LAIV has been shown to induce T cell responses within the lungs of mice following whole lung inoculation (6), when LAIV vaccination has been limited to the upper respiratory tract in animal models, similar to its replication location in humans, it does not induce T cell responses within the lower lung mucosa (7).

Many recent efforts at “universal” vaccination have been focused on targeting the antibody response toward the more conserved stem region of the hemagglutinin (HA) IAV protein (8, 9). However, infection-induced immunity also confers protection through underlying T cell responses that can provide cross-strain protection. T cell-mediated heterosubtypic protection has been well described in animal models (10–13) and was shown to confer increased protection in humans during the most recent 2009 H1N1 pandemic (12). Furthermore, studies in animal models of IAV infection have demonstrated that the pulmonary immune system imprints effector T cells with lung homing capabilities as well as induces the formation of local tissue-resident memory T and B cells that are thought to provide optimal protection (13–18). This tissue-resident phenotype is thought to depend on antigen longevity, antigen presenting cells (APC), and tertiary structures within the tissues (18–23). Therefore, vaccines that utilize tissue-specific factors and pathways critical for the induction of pulmonary T and B cell responses to generate local as well as systemic immunity by mimicking IAV infection would be predicted to confer more robust protection.

We have previously reported a novel polyanhydride [copolymers of 1,8-bis(*p*-carboxyphenoxy)-3,6-dioxoctane (CPTEG) and 1,6-bis(*p*-carboxyphenoxy)hexane (CPH)] nanoparticle-based vaccine platform that has shown great promise in inducing immunity when administered subcutaneously (s.c.) (24–26). This platform offers several distinct advantages: the particles degrade into biocompatible products, activate APC, maintain the stability of encapsulated antigen, enable dose sparing of the antigen, and may be stored at room temperature or higher for up to 4 months thus breaking the cold chain (27, 28). Another important feature of our nanoparticle technology is that it provides a sustained release of encapsulated antigen via surface erosion and acts as a long-term antigen depot. Therefore, it could mimic the antigen depot that occurs after IAV infections and potentiate tissue-resident memory formation (21, 22, 29). However, the capability of intranasal (i.n.) administration of polyanhydride nanoparticles to induce local (i.e., lung) and systemic adaptive immunity, drive tissue-resident memory formation, and offer cross-strain protection against IAV has not yet been explored.

To this end, we tested the immune capabilities of an i.n. administered CPTEG:CPH polyanhydride nanovaccine containing HA and nucleocapsid protein (NP) proteins from an H1N1 strain (A/Puerto Rico/8/1934) of IAV and CpG 1668, hereafter referred to as IAV-nanovax. Our results illustrate that i.n. vaccination with IAV-nanovax induced robust lung-resident germinal center (GC) B cells along with systemic and lung localized IAV-specific antibody responses. Notably, similar to IAV infections, i.n. administered IAV-nanovax induced lung-resident memory CD4 and CD8 T cell responses. These IAV-specific humoral and cellular immune responses were associated with protection against homologous and heterologous infection as vaccinated mice were protected against subsequent lethal dose challenges with H1N1 and H3N2 strains of IAV, respectively.

## Materials and methods

### IAV-nanovax synthesis

Monomers based on 1,8-bis(*p*-carboxyphenoxy)-3,6-dioxoctane (CPTEG) and 1,6-bis(*p*-carboxyphenoxy)hexane (CPH) were synthesized as described previously (30, 31) Using these monomers, 20:80 CPTEG:CPH copolymer was synthesized using melt polycondensation for ~6 h, as described (31). The final copolymer composition, purity, and molecular weight of the copolymer were characterized using ^1^H HNMR (DXR 500, Bruker, Billerica, MA). Next, 20:80 CPTEG:CPH nanoparticles containing 1% H1 HA, 1% NP, and 2% CpG1668 were synthesized via solid-oil-oil double emulsion (32). Briefly, HA and NP protein antigens (Sino Biological, Beijing, China) were dialyzed to nanopure water and lyophilized overnight. The 20:80 CPTEG:CPH copolymer, along with HA, NP, and CpG (ODN 1668, Invivogen, San Diego, CA), was dissolved at a polymer concentration of 20 mg/mL in methylene chloride. The solution was sonicated for 30 s and then precipitated into chilled pentane (at a methylene chloride:pentane ration of 1:250). The resulting nanoparticles were collected via vacuum filtration and scanning electron microscopy (FEI Quanta 250, FEI, Hillsboro, OR) was used to characterized morphology and size.

### Mice, vaccination, and influenza virus infection

Wild type female C57BL/6 mice were bred, housed, and maintained in the University of Iowa (Iowa City, IA) animal care facilities. Swiss-Webster mice (NCI Cr:SwWEB) were purchased from Charles River Laboratories, Inc (Frederick, MD) and maintained in the University of Iowa (Iowa City, IA) animal care facilities. All procedures were performed on matched mice, were approved by the Institutional Animal Care and Use Committee of the University of Iowa and comply with the NIH Guide for Care and Use of Laboratory Animals. Mice were randomly assigned into groups for each experiment.

Prior to i.n IAV-nanovax vaccinations and IAV infections, mice were anesthetized with isoflurane. For each IAV-nanovax i.n. administration, mice received 500 μg of IAV-nanovax (containing a total of 5 μg HA + 5 μg NP + 10 μg CpG1668) in 50 μL of PBS containing 2.5 μg each of free HA and NP proteins. In prime+boost experiments, mice received a second i.n. dose of IAV-nanovax 14 days after the initial IAV-nanovax priming. For those experiments utilizing IAV-nanovax vaccination without the free antigen component, mice received i.n. 500 μg of IAV-nanovax (containing a total of 5 μg HA + 5 μg NP + 10 μg CpG1668) in 50 μL of PBS followed by a second i.n. dose of IAV-nanovax without free antigen 14 days after the initial IAV-nanovax priming. For those experiments utilizing vaccination with polyanhydride particles that only contained CpG1668 (CpG Particles), mice received i.n. 500 μg of CpG Particles in 50 μL of PBS. For IAV infections, mice were infected i.n. with a 110 TCIU or 1108 TCIU dose of mouse adapted A/Puerto Rico/8/34 (H1N1) or a 390 TCIU dose of A/Hong Kong/1/68 (H3N2) strains in 50 μL Iscove's Modified Dulbecco's Medium. After infection mice were euthanized upon reaching 70% of their starting weight. For IIV vaccinations, non-anesthetized mice received either one dose or two doses separated by 14 days of 20 μg of beta-propiolactone inactivated A/Puerto Rico/8/34 (H1N1) IAV in 200 μL of PBS i.m. in the caudal thigh muscle.

### Measurement of airway resistance

Enhanced pause (Penh), an indicator of lung function (i.e., airway resistance), was measured using unrestrained whole-body plethysmography (Buxco Electronics, Wilmington, NC) on non-anesthetized mice as previously described (33). Penh values were recorded daily based on volume and pressure changes over 5 min.

### Measurement of influenza virus titers

Lung viral titers were analyzed by plaque assay on whole lung homogenates. Briefly, serial dilutions of homogenized lung samples were applied to confluent Madin-Darby canine kidney epithelial cell layers and incubated for 1 h at 37°C. Cell layers were washed and a minimum essential media agar overlay was applied and incubated for 3 days at 37°C. Cell layers were fixed in 4% formaldehyde, blocked with 5% milk, and plaques were detected with polyclonal anti-IAV A/Puerto Rico/8/34 (H1N1) chicken antiserum (NR-3098; BEI Resources), peroxidase-conjugated AffiniPure rabbit anti-chicken IgY (Jackson Immunoresearch, West Grove, PA), and TrueBlue® peroxidase substrate (KPL, Gaithersburg, MD).

### Intravascular stain to determine cellular localization

Three minutes prior to euthanasia, mice were administered 1 μg of fluorophore-conjugated rat anti-mouse CD45.2 (clone 104; BioLegend, San Diego, CA) in 200 μL of PBS by retroorbital intravenous injection as previously described (34).

### Serum, bronchial alveolar lavage, and cell isolation

Prior to euthanasia, blood was collected in heparinized capillary tubes (Fisher Scientific, Pittsburgh, PA) for subsequent single-cell analysis by flow cytometry and non-heparinized capillary tubes (Fisher Scientific) for serum collection. For cell harvests, these blood samples were then treated with ammonium-chloride-potassium lysis buffer for 5 min at room temperature and washed 1X with flow cytometry staining buffer. For serum collection, blood samples were left at room temperature for 30 min, centrifuged at 16,000 × g for 20 min, and then collected and stored at −20°C until analysis.

Bronchial alveolar lavage (BAL) fluid was collected using a protocol modified from (35). Briefly, the tracheae were cannulated with a 22-gage catheter tube (attached to a 5cc syringe) and then washed once with 1 mL of sterile PBS. Samples were stored at −20°C until analysis.

For preparation of cells from lungs and spleens, these organs were harvested after the collection of BAL fluid, digested for 30 min at 37°C in media containing 1 mg/mL Collagenase (Type 3; MP Biomedicals, Solon, OH) and 0.02 mg/mL DNase-I (MP Biomedicals), and then pressed through wire mesh to obtain a single cell suspension.

### IAV-specific whole virus ELISAs

Total IAV-specific IgG and IgA antibody against whole A/Puerto Rico/8/34 live virus was measured as previously described (36). Briefly, wells were coated with ~3.2 × 10^5^ TCIU_50_ of virus, blocked with 1% bovine serum albumin, washed, and then blotted dry. Serum or BAL samples were added to the top well in triplicate at a 1:50 or 1:4 dilution in 200 μL/well, respectively. Samples were serially diluted at 1:2 and incubated at 37°C for 2 h. Plates were washed, blotted dry, and then IAV-specific antibody was detected using the following antibodies: biotin-labeled goat anti-mouse IgA (Southern Biotechnology Associates, Birmingham, AL); biotin-labeled AffiniPure goat anti-mouse IgG, Fc fragment specific (Jackson Immunoresearch Laboratories) followed by alkaline phosphatase-streptavidin (Invitrogen, Carlsbad, CA) and 2 mg/mL phosphatase substrate (Sigma-Aldrich, St. Louis, MO). Optical densities were measured at 405 nm using SpectraMax M5 Multi-mode microplate reader from Molecular Devices (Sunnyvale, CA).

### Hemagglutination inhibition assay

Hemagglutination inhibition (HAI) assays using mouse serum and BAL were performed as previously described (37). Briefly, sera and BAL were inactivated by heating at 56°C for 30 min and then absorbed in a chicken red blood cell (CRBC) suspension for 30 min at different concentrations: serum was absorbed in 1% CBRC at 1:5 and BAL was absorbed in 10% CBRC at 1:2. CBRCs were pelleted and both sera and BAL were serial diluted in 96-well round-bottom plates that were then incubated with four hemagglutination units of stock virus per well for 30 min. Each well then received 1% CBRC suspension and HAI titer was measured after a 30 min incubation.

### Antibody staining for flow cytometry

Single-cell suspensions (1 × 10^6^ cells) from lungs were blocked with 2% rat serum for 30 min at 4°C. Following blocking, cells were stained with the following antibodies: rat anti-mouse CD4 (GK1.5; BioLegend), rat anti-mouse CD8α (53-6.7; BioLegend), rat anti-mouse CD49d (R1-2, BioLegend), rat anti-mouse CD11a (M17/4; BD Biosciences, San Jose, CA), rat anti-mouse CD103 (M290; BD Biosciences), and rat anti-mouse CD69 (H1.2F3; eBioscience), to identify CD4 and CD8 T cell subsets. Antigen experienced T cells were identified via expression of surrogate markers as previously described (38, 39). Briefly, CD11a^hi^CD49d^pos^ expression was utilized to identify antigen-experienced CD4 T cells, while CD11a^hi^CD8α^lo^ expression was utilized to quantify antigen-experienced CD8 T cells. To identify B cell subsets, cells were stained with rat anti-mouse CD19 (1D3; BD Biosciences), rat anti-mouse B220 (RA3-6B2; BioLegend), rat anti-mouse IgM (B7-6), and FITC-conjugated peanut agglutinin (PNA; Vector Laboratories, Burlingame, CA). Cells were then fixed with BD FACS™ Lysing Solution per manufacturer's instructions and resuspended in PBS. Data were acquired on a LSRII (BD Biosciences) and analyzed using FlowJo software (Tree Star, Ashland, OR).

### Statistical analysis

Experiments were repeated at least twice unless noted otherwise. Comparisons between two groups was performed with a two-tailed student's *t-*test. Comparisons between more than two groups at different time points were analyzed using two-way ANOVA with Holm–Sidak's multiple comparison *post-hoc* test. For comparisons between more than two groups at a single time point, a D'Agostino and Pearson normality test was performed to establish normality. Data that failed normalcy were analyzed using a Kruskal–Wallis ANOVA with a Dunn's multiple comparison *post-hoc* test. Data that passed normalcy were analyzed using a one-way ANOVA with a Tukey's multiple comparison *post-hoc* test. A *P* ≤ 0.05 was considered significant.

## Results

### IAV-nanovax induces lung-resident GC B cells and IAV-specific antibody responses

In order to design an IAV vaccine that provides optimal protection by inducing long-lived local (i.e., lungs) and systemic immune responses, we made use of our CPTEG:CPH polyanhydride nanovaccine platform. Our previous studies have shown that a 20:80 CPTEG:CPH copolymer-based nanoparticle formulation is an effective delivery vehicle for IAV antigens and generation of systemic immune responses when given s.c. (26). Therefore, in order to generate both lung-focused as well as systemic immunity, we designed an i.n. vaccine (IAV-nanovax) consisting of 20:80 CPTEG:CPH nanoparticles encapsulating 5 μg of both IAV HA and NP proteins [source A/Puerto Rico/8/34 (H1N1)] along with a 10 μg CpG oligo (ODN 1668) that is known to induce cross-presentation by dendritic cells (40). The HA protein was included as it is a primary component of current vaccination strategies and is a focus of neutralizing antibody responses. In addition, NP protein was incorporated as it has been shown to drive NP-specific T cell responses that provide protection against heterologous infection as well as induce non-neutralizing antibody responses that facilitate more rapid T cell responses upon subsequent exposures (41, 42). These nanoparticles were then administered i.n. in water along with 2.5 μg of free HA and NP proteins in a prime+boost regimen as previous work from our laboratories (25, 26) has shown that the additional soluble antigen together with the nanovaccine during a prime+boost vaccination enhanced the immune response and protection following subcutaneous vaccination.

**Figure 1:**
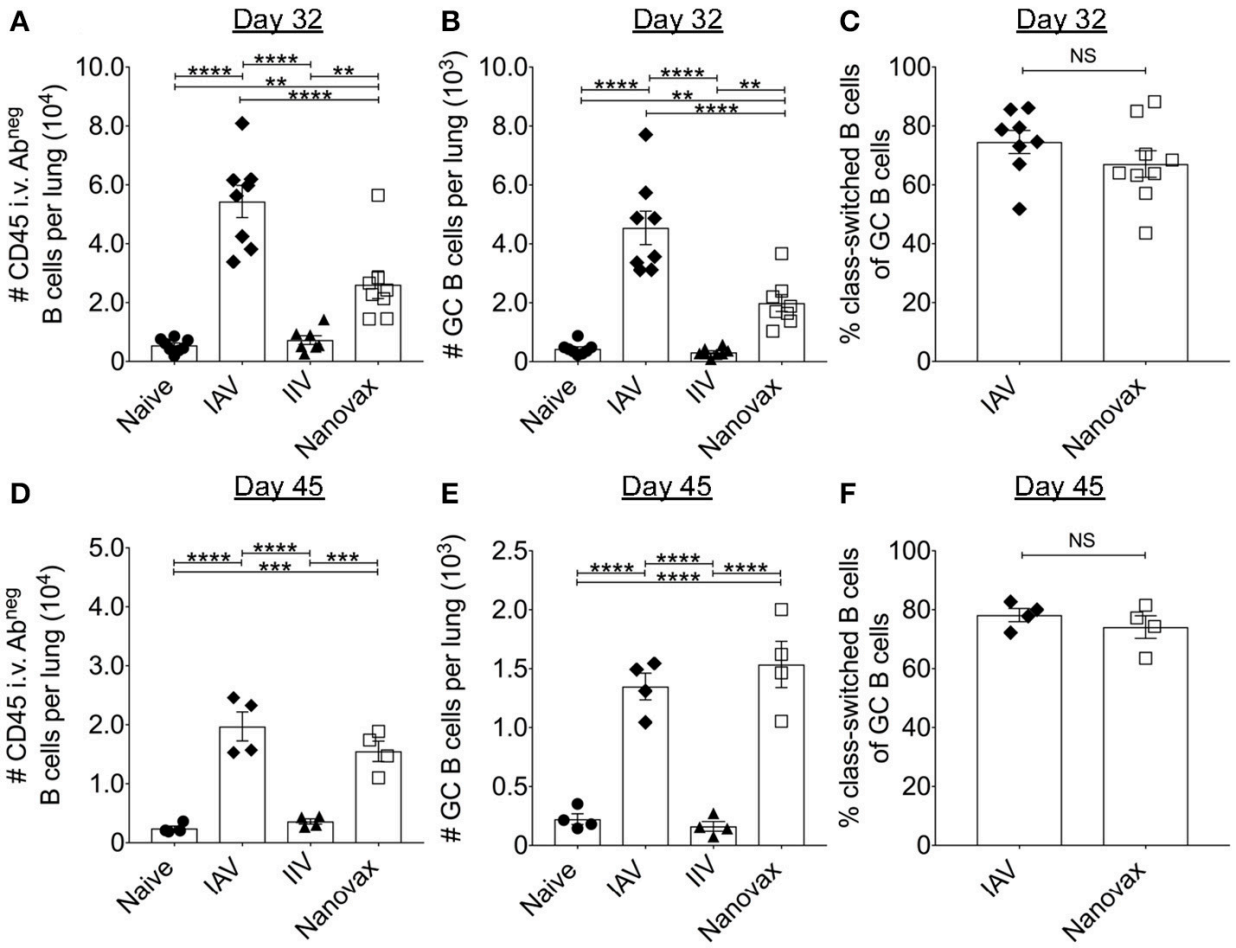
Vaccination with IAV-nanovax induces lung-resident germinal center B cell responses. C57BL/6 mice were challenged i.n. with a 110 TCIU of A/Puerto Rico/8/1934, vaccinated i.m. with IIV, prime+boost vaccinated i.n. with IAV-nanovax (Nanovax), or left unchallenged/unvaccinated (naïve). At 32 and 45 days post challenge/vaccination, **(A,D)** lung-resident B cells, **(B,E)** germinal center (GC) B cells, and **(C,F)** class switched B cells were enumerated within the lungs. Error bars, mean ± s.e.m. Data are from two pooled experiments (**A–C**; *n* = 8 mice/group) or one (**D–F**; *n* = 4 mice/group) independent experiment. ^**^P < 0.01, ^***^P < 0.001, ^****^P < 0.0001 (One-way ANOVA with Tukey's multiple comparisons test).

Since the generation of IAV-specific antibody responses are frequently used to determine IAV vaccine efficacy, we began by analyzing B cell responses in the lungs following i.n. IAV-nanovax vaccination and compared the response to mice i.n. infected with IAV (PR8; H1N1), mice i.m. vaccinated with IIV, or mice that were left untreated (naïve). In order to distinguish between lymphocytes embedded in the lung interstitium from those in the vasculature, we utilized an *in vivo* fluorophore-conjugated antibody labeling technique (34) (Supplemental Data Sheet 1). To this end, mice were intravascularly (i.v.) infused prior to lung harvest with a fluorescent antibody to label B cells within the circulation (CD45i.v.Ab^pos^) vs. those in the lung parenchyma (CD45i.v.Ab^neg^) (Supplemental Data Sheet 1). Using this technique, we observed that total lung-resident B cells (CD19^pos^B220^pos^CD45i.v.Ab^neg^) were significantly higher for IAV-infected and IAV-nanovax vaccinated mice compared to naïve and IIV controls at 32 and 45 days following infection/vaccination (Figures 1A,D). Consistent with the increase in total numbers in IAV-infected and IAV-nanovax treated mice, lung-resident GC B cells (CD19^pos^B220^pos^CD45i.v.Ab^neg^PNA^pos^) were also significantly elevated at both time points (Figures 1B,E and Supplemental Data Sheet 1). Similar trends were also observed in the lung draining lymph nodes (data not shown). As GC B cell reactions result in class-switched B cells that produce higher affinity antibodies, we next compared the frequencies of IgM^neg^ lung-resident GC B cells between IAV-infected and IAV-nanovax treated mice. As previously shown, IAV infection induces a substantial proportion of the GC response in the lungs to switch to IgG (IgM^neg^IgG^pos^) (36). Similar to IAV-infected mice, approximately 70% of lung resident GC B cells were IgM^neg^ in IAV-nanovax mice, a finding consistent with a robust, mature GC response (Figures 1C,F). These results suggest that i.n. administration of IAV-nanovax induces lung-resident GC B cell responses capable of producing class-switched B cells to levels commensurate to those found in IAV-infected mice at 45 days following infection/vaccination.

**Figure 2:**
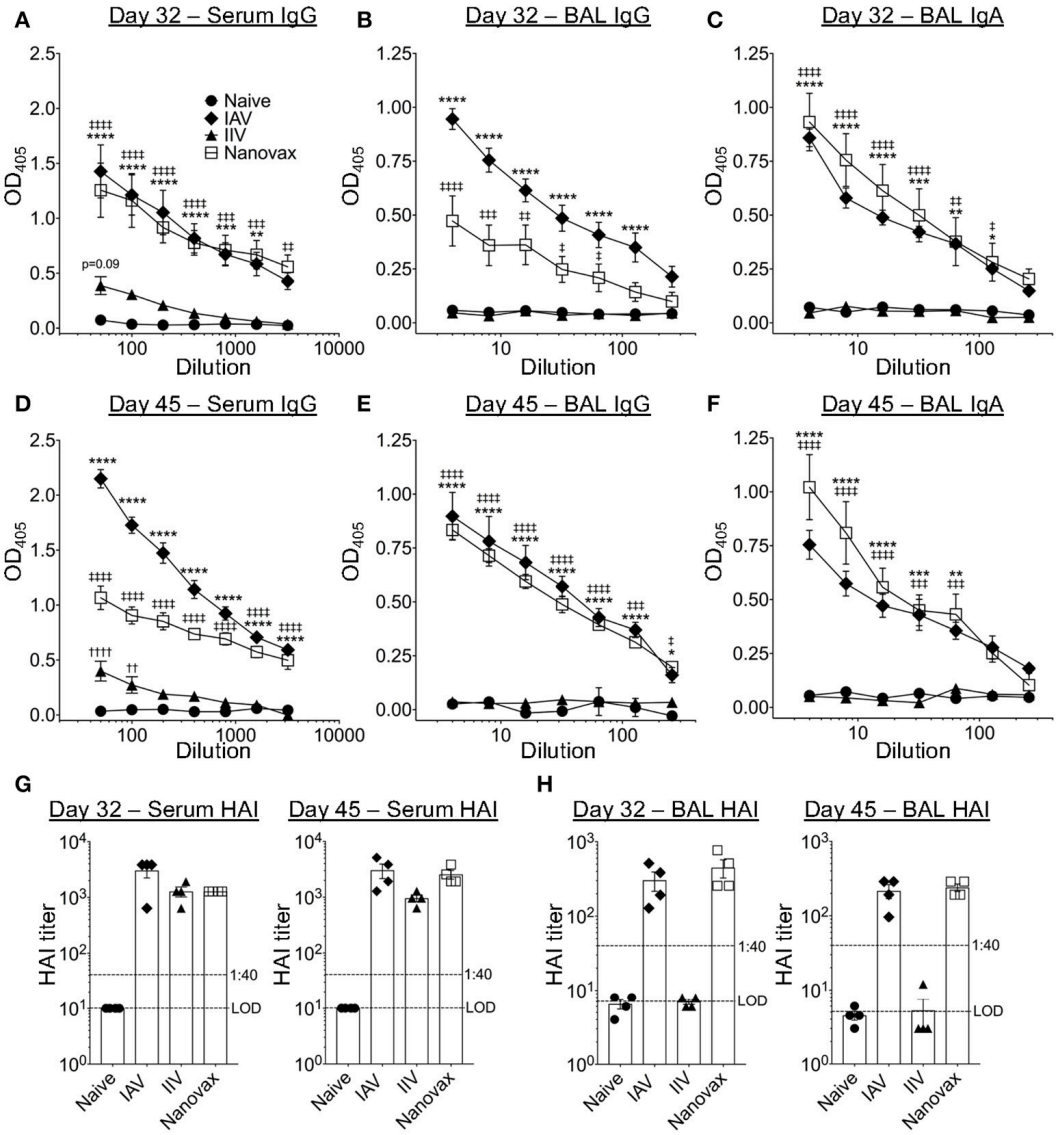
IAV-nanovax vaccination induces both lung and systemic IAV-specific antibody responses. C57BL/6 mice were vaccinated/infected as described in Figure 1. At 32 and 45 days post challenge/vaccination, serum and BAL were collected. Total IAV-specific serum IgG **(A,D)**, BAL IgG **(B,E)**, and BAL IgA **(C,F)** were quantified by ELISA. Serum **(G)** and BAL **(E)** HAI levels were quantified. Error bars, mean ± s.e.m. LOD, limit of detection. Data are representative of three independent **(A–C, G)** or two independent **(D–F, H)** experiments with *n* = 4–5 mice/group. IAV vs. naïve: ^*^P < 0.05, ^**^P < 0.01, ^***^P < 0.001, ^****^P < 0.0001; Nanovax vs. naïve: ‡*P* < 0.05, ‡‡*P* < 0.01, ‡‡‡*P* < 0.001, ‡‡‡‡*P* < 0.0001; IIV vs. naïve: ^*††*^*P* < 0.01, ^*††††*^*P* < 0.0001 (Two-way ANOVA with Holm-Sidak multiple comparisons test).

To determine if the observed B cell responses generated IAV-specific antibodies, we quantified total IAV-specific IgG and IgA antibody following infection or vaccination. As expected, IAV-specific IgG responses were detected locally [i.e., bronchoalveolar lavage (BAL)] and systemically (i.e., serum) in IAV-infected and IAV-nanovax vaccinated mice at 32 and 45 days following infection/vaccination (Figures 2A,B,D,E). Interestingly, serum levels of IAV-specific IgG antibodies were ~2–3x higher in animals after IAV-nanovax and IAV infection than in mice receiving IIV (Figures 2A,D). Mice receiving IIV also lacked robust IAV-specific IgG within the BAL as observed in IAV-nanovax and IAV infected mice (Figures 2B,E). This difference in measurable IAV-specific IgG within the BAL between IIV and IAV-nanovax is likely related to the lack of a local lung GC response in the IIV vaccinated mice (Figure 1). Previous studies have demonstrated that IgA is present in the BAL after IAV infection (36). Consistent with this idea we found that both IAV-infection and IAV-nanovax, but not IIV, induced IAV-specific IgA levels in the BAL (Figures 2C,F). To determine the potential of these IAV-specific antibodies to contribute to protection from lethal dose IAV infections, we measured the capability of serum and BAL antibodies to inhibit IAV-hemagglutination. At 32 and 45 days post infection/vaccination, both IIV and IAV-nanovax vaccinated mice had serum hemagglutination inhibition (HAI) titers that were similar to IAV infected mice and well above the >1:40 mark that is associated with protection against subsequent IAV infection (Figures 2G) (43). However, only IAV infected and IAV-nanovax vaccinated mice possessed protective levels of HAI antibodies within the BAL (Figure 2H). Altogether, these results suggest that i.n. administration of IAV-nanovax induces both local and systemic IAV-specific antibody responses capable of providing protection against IAV infection.

### IAV-nanovax generates antigen experienced CD4 and CD8 T cell responses within the lungs

**Figure 3:**
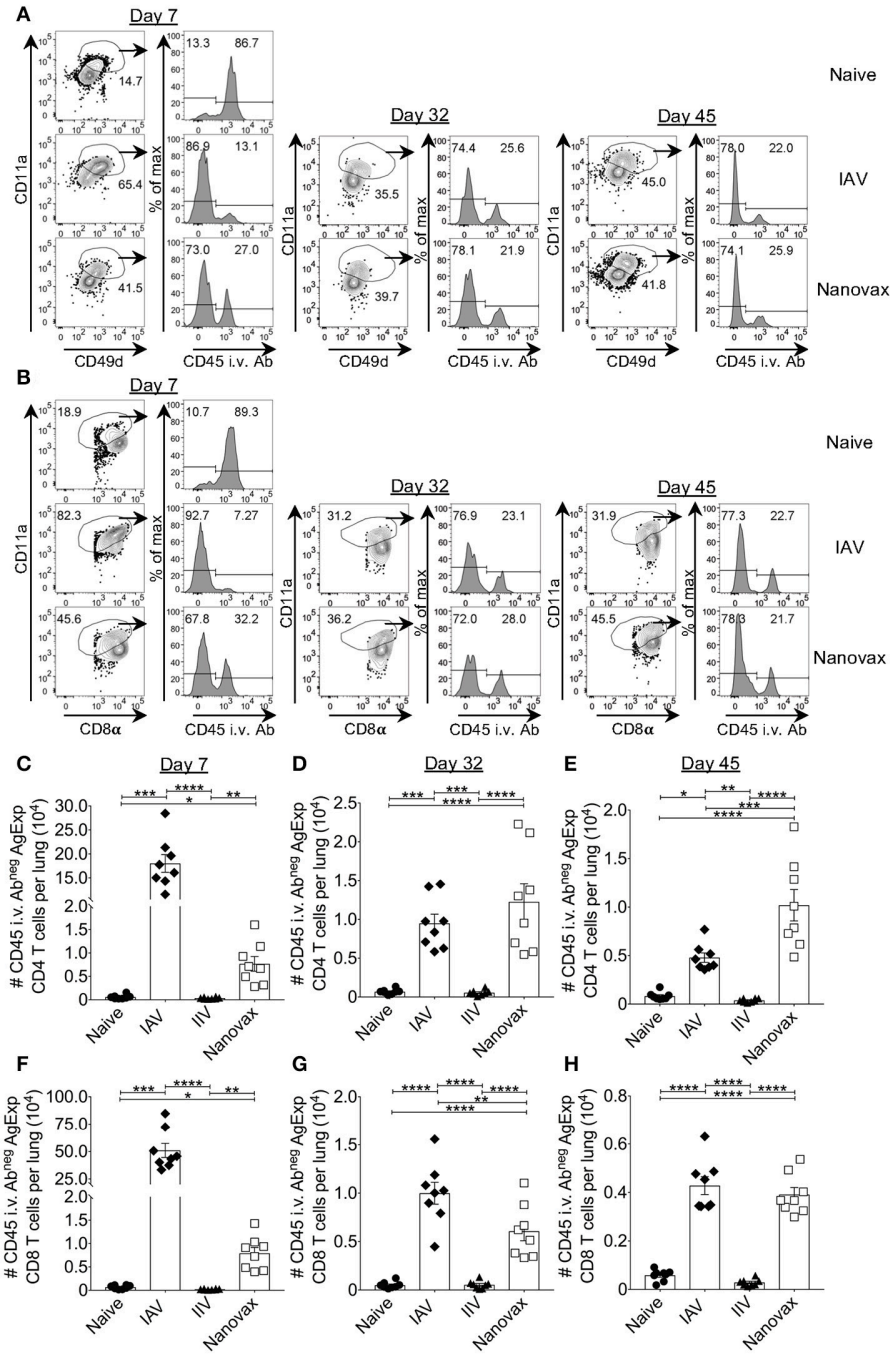
IAV-specific lung-resident CD4 and CD8 T cell responses are induced following IAV-nanovax vaccination. C57BL/6 mice were vaccinated/infected as described in Figure 1. At day 7, 32, and 45 post challenge/vaccination lungs were harvested. Representative gating strategies for **(A)** lung-resident AgExp CD4 T cells (CD11a^hi^CD49d^pos^CD45 i.v.Ab^neg^) and **(B)** lung-resident AgExp CD8 T cells (CD11a^hi^CD8α^lo^CD45 i.v.Ab^neg^). Numbers of **(C–E)** lung-resident AgExp CD4 and **(F–H)** lung-resident AgExp CD8 T cells were determined. Error bars, mean ± s.e.m. Data are from two pooled experiments with *n* = 8 mice. ^*^P < 0.05, ^**^P < 0.01, ^***^P < 0.001, ^****^P < 0.0001 (Day 7, Kruskal–Wallis ANOVA with Dunn's multiple comparisons test; Day 32 and 45, One-way ANOVA with Tukey's multiple comparisons test).

Generation of GC B cell responses and class-switched antibodies are often associated with antigen-specific CD4 T cell responses. It has also been shown that IAV-specific CD8 T cells are important for control of IAV. Therefore, we determined the capacity of IAV-nanovax to elicit IAV-specific CD4 and CD8 T cell responses within the lungs. The CD4 T cell response to IAV has been shown to encompass a large number of epitopes, each only being expressed at low frequency (44). Thus, in order to not bias the response by examining a single epitope specificity we utilized a surrogate marker staining strategy. This strategy identifies total antigen-experienced T cells (Figures 3A,B), including those where epitopes have not been identified or are limited (38, 39). Compared to naïve mice, IAV infection and IAV-nanovax vaccination generated an increased frequency of antigen-experienced CD4 T cells (AgExp CD4; CD4^pos^CD11a^hi^CD49d^pos^) and antigen-experienced CD8 T cells (AgExp CD8; CD8^lo^CD11a^hi^) within the lungs at days 7, 32, and 45 (Figures 3A,B). We further observed that a vast majority of these AgExp CD4 and AgExp CD8 T cells were resident within the lung tissue (CD45i.v.Ab^neg^) based on *in vivo* antibody labeling in IAV-nanovax vaccinated mice, similar to that observed following IAV-infection (Figures 3A,B). Importantly, these lung-resident AgExp CD4 and CD8 T cells in IAV-nanovax vaccinated mice were found in higher numbers compared to naïve and IIV vaccinated mice at 7, 32, and 45 days post vaccination (Figures 3C–H). Although numbers of lung-resident AgExp CD4 and CD8 T cells were higher early (day 7) in IAV-infected mice compared to IAV-nanovax mice (Figures 3C,F), IAV-nanovax was capable of inducing T cell responses of a similar magnitude to those observed in the IAV infected lung at later time points (Figures 3D,E,G,H)

### Lung-resident CD4 and CD8 T cells generated following IAV-nanovax vaccination have a memory phenotype

**Figure 4:**
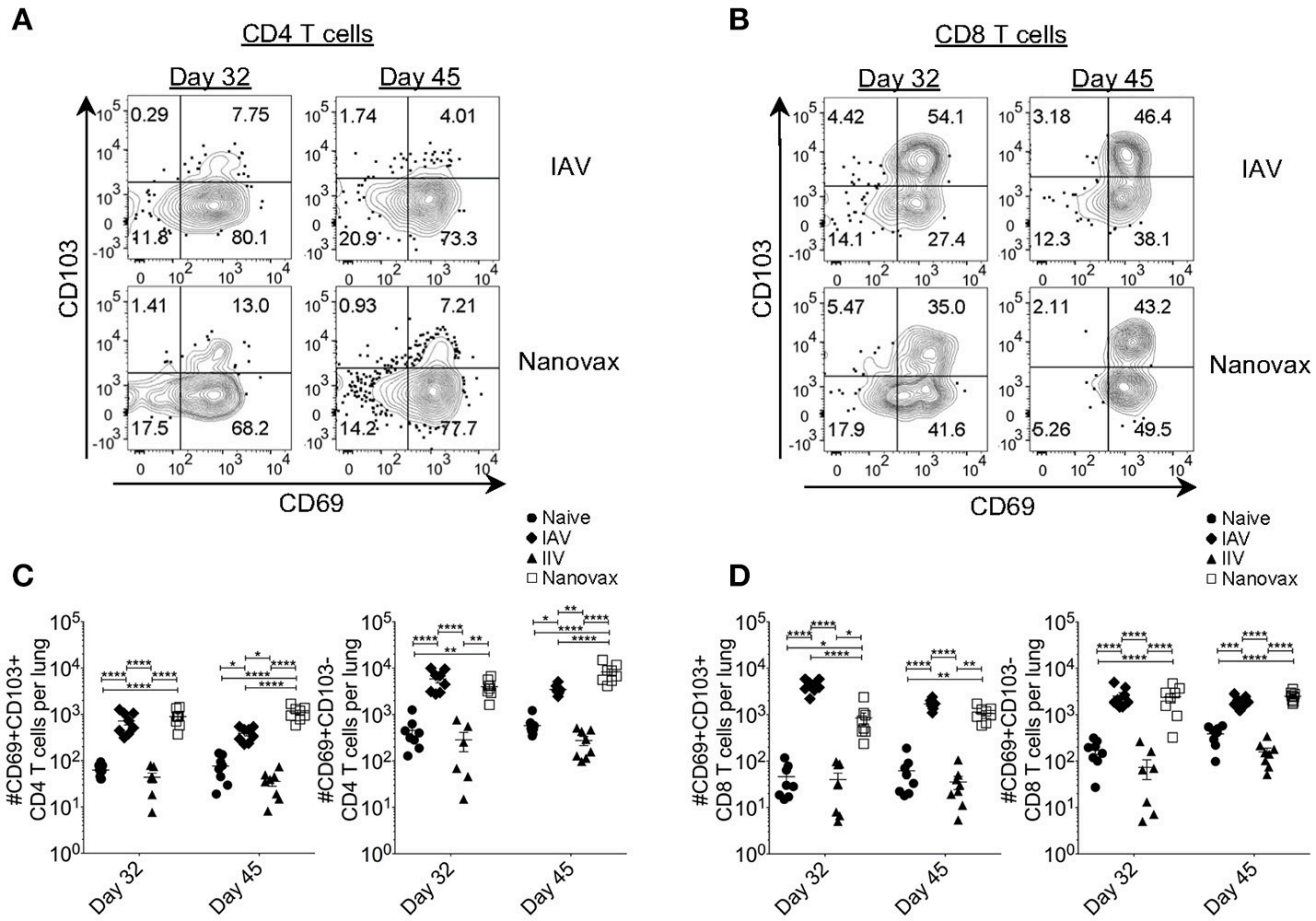
Vaccination with IAV-nanovax induces IAV-specific tissue-resident memory CD4 and CD8 T cells within the lungs. C57BL/6 mice were vaccinated/infected as described in Figure 1. At 32 and 45 days post challenge/vaccination, **(A)** lung-resident AgExp CD4 T cells and **(B)** lung-resident AgExp CD8 T cells were characterized for their expression of CD69 and CD103. Total numbers of **(C)** lung-resident memory CD4 T cells and **(D)** lung-resident memory CD8 T cells were determined. Error bars, mean ± s.e.m. Data are two pooled experiments with *n* = 8 mice/group. ^*^P < 0.05, ^**^P < 0.01, ^****^P < 0.0001 (One-way ANOVA with Tukey's multiple comparisons test).

Recent studies have demonstrated that the presence of lung-resident memory T cells after IAV infection increases protection (13, 15–18). Therefore, we next determined whether the robust lung-resident AgExp CD4 and CD8 T cell responses generated by IAV-nanovax vaccination shared phenotypic characteristics with canonical lung-resident memory T cells (Trm). In the IAV-infected lung, the expression of CD69 was prominent in lung-resident (i.e., CD45i.v.Ab^neg^) AgExp CD4 T cells at 32 and 45 days following IAV infection, a change that is associated with establishment of lung-resident memory cells (45) (Figure 4A). This trend was also observed in IAV-nanovax vaccinated mice; however, IAV-nanovax induced a greater fraction of canonical CD69^pos^ AgExp CD4 Trm cells as well as a subset that co-expressed CD103 (Figures 4A,C). Nevertheless, both the CD69^pos^CD103^pos^ and CD69^pos^ CD103^neg^ lung-resident AgExp CD4 T cell subsets were elevated in IAV-nanovax, but not IIV vaccinated, mice to levels equal to or higher than those observed in IAV-infected mice (Figure 4C). In contrast to lung Trm CD4 T cells, lung Trm CD8 T cells have been reported to co-express CD69 and CD103 (46). By day 32 following IAV-infection or IAV-nanovax vaccination, the fraction and number of CD69^pos^CD103^pos^ AgExp CD8 T cells resident within the lungs were significantly increased relative to naïve and IIV vaccinated mice (Figures 4B,D). Albeit the number of CD8 Trm were initially higher in IAV-infected mice, IAV-nanovax vaccinated mice exhibited similar CD8 Trm responses to IAV-infected mice by day 45 post infection/vaccination (Figure 4D). Overall, these data suggest that IAV-nanovax vaccination induces CD4 and CD8 Trm responses of similar magnitudes to IAV infection.

### IAV-nanovax provides protection against homologous and heterologous IAV infections

**Figure 5:**
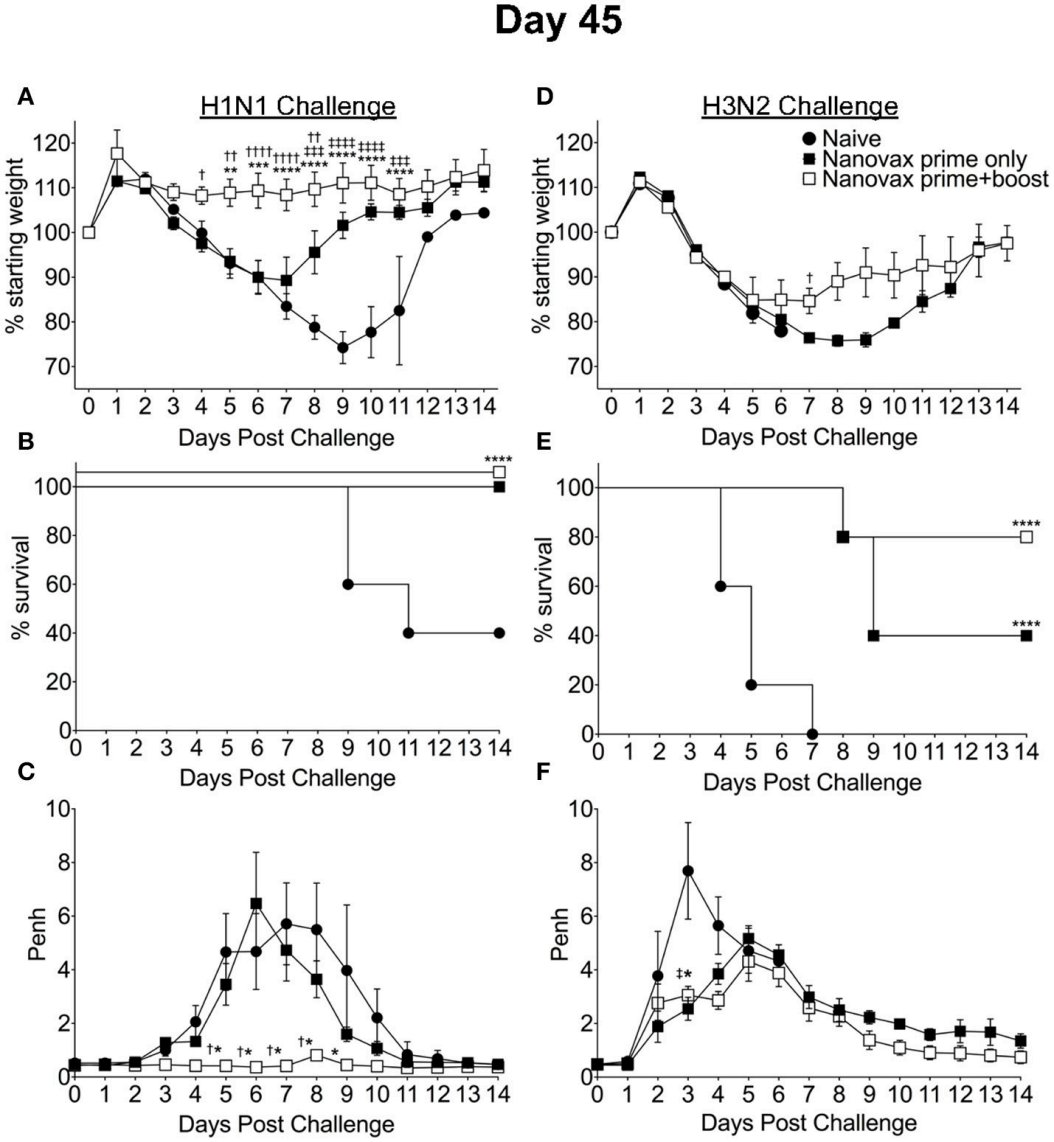
IAV-nanovax confers protection against subsequent homologous and heterologous IAV infection. C57BL/6 mice received one-dose i.n. of IAV-nanovax (prime only), two-doses i.n. of IAV-nanovax (prime+boost), or were left unvaccinated (naive). Forty-five days following the initial vaccination, mice were challenged with a **(A–C)** 1108 TCIU dose of A/Puerto Rico/8/1934 (H1N1) or **(D–F)** 390 TCIU dose of A/Hong Kong/1/1968 (H3N2). Morbidity and mortality were measured by daily weight loss **(A,D)** and survival **(B,E)**. **(C,F)** Penh was recorded daily as a measurement of lung function (airway resistance). Error bars, mean ± s.e.m. Data are representative of two independent **(C,F)** or three independent **(A,B,D,E)** experiments with *n* = 5 mice/group. **(A,C,D,F):** Nanovax prime+boost vs. naïve: ^*^P < 0.05, ^**^P < 0.01, ^***^P < 0.001, ^****^P < 0.0001; Nanovax prime only vs. naïve: ‡*P* < 0.05, ‡‡‡*P* < 0.001, ‡‡‡‡*P* < 0.0001; Nanovax vs. IAV: ^*†*^*P* < 0.05, ^*††*^*P* < 0.01, ^*††††*^*P* < 0.0001 (Two-way ANOVA with Holm–Sidak multiple-comparison test). **(B,E)** ^****^P = 0.0001 to naïve (Mantel-Cox Log rank test).

Given the robust pulmonary B and T cell responses we observed following IAV-nanovax vaccination, we next determined the potential of IAV-nanovax to circumvent IAV associated morbidity and mortality upon subsequent exposures. Further, since IAV-nanovax induced pulmonary CD4 and CD8 T cell responses within the lungs by day 7 post vaccination (i.e., prior to the boost, Figure 3) we additionally compared protection after a prime only vs. a prime+boost vaccination schedule. Forty-five days after the initial vaccination, mice were challenged with a lethal dose of homologous IAV (A/Puerto Rico/8/34). As expected, naïve mice displayed substantial disease associated weight-loss (>20%), mortality (60%), and respiratory distress, as measured by increases in airway resistance (~6 Penh); however, mice that received either prime only or prime+boost IAV-nanovax administration exhibited reduced signs of morbidity and were completely protected against mortality (Figures 5A–C). This alleviation of disease is commensurate to IIV vaccinated mice as similar trends of reduced morbidity and mortality were also observed for IIV prime+boost vaccinated mice compared to naïve (Supplemental Data Sheet 2A–C). Strikingly, IAV-nanovax prime+boost mice exhibited little to no weight loss or increases in Penh demonstrating that this strategy provides more robust protection compared to prime only mice (Figures 5A,C). Consistent with this disease amelioration, the IAV-nanovax prime+boost mice had significantly reduced lung viral titers 3 days following challenge indicating early control of viral replication (Supplemental Data Sheet 3).

**Figure 6:**
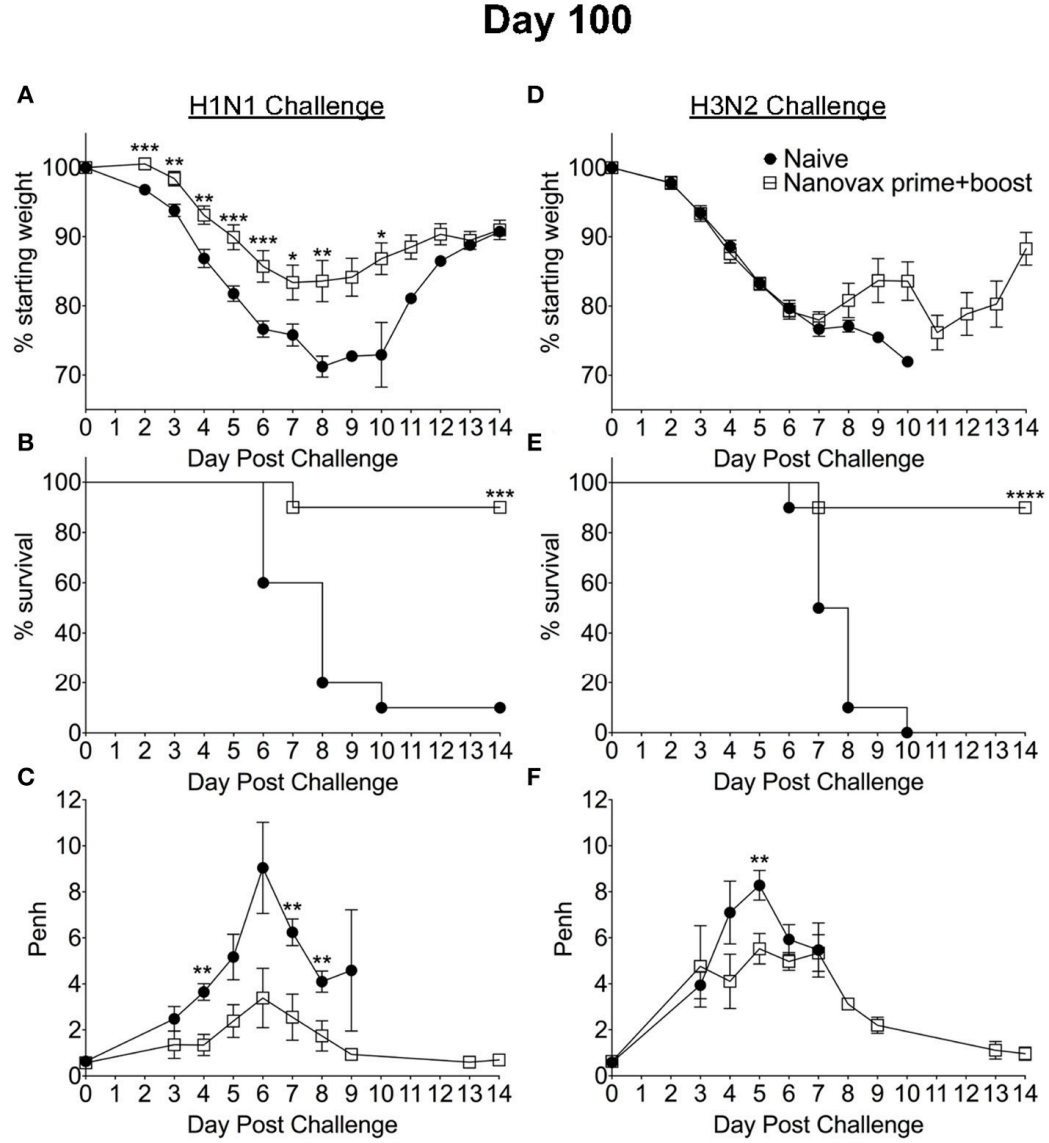
Homologous and heterologous protection mediated by IAV-nanovax is long-lived. C57BL/6 mice received two-doses i.n. of IAV-nanovax (prime+boost) or were left unvaccinated (naive). One-hundred days following the initial vaccination, mice were challenged with a **(A–C)** 1108 TCIU of A/Puerto Rico/8/1934 (H1N1) or **(D–F)** 390 TCIU of A/Hong Kong/1/1968 (H3N2). Morbidity and mortality were measured by daily weight loss **(A,D)** and survival **(B,E). (C,F)** Penh was recorded daily as a measurement of lung function (airway resistance). Error bars, mean ± s.e.m. Data are of two pooled experiments **(A,B,D,E)** with *n* = 10 mice/group or representative of one independent experiment **(C,F)** with *n* = 5 mice/group. **(A,C,D,F)** ^*^P < 0.05, ^**^P < 0.01, ^***^P < 0.001 (Two-tailed student's *t*-test). **(B,E)** ^***^P < 0.001, ^****^P < 0.0001 (Mantel-Cox Log rank test).

Since IAV-nanovax generated robust CD4 and CD8 Trm responses and recent studies have emphasized the importance of lung-resident CD4 and CD8 memory T cells in providing protection against subsequent heterologous IAV infections (13, 15–18), we next determined if IAV-nanovax vaccination could confer protection against a heterologous IAV challenge. To this end, prime only and prime+boost IAV-nanovax vaccinated mice were challenged with a lethal dose of a mouse-adapted heterologous strain of IAV (A/Hong Kong/68, H3N2). Early (days 1–5) following challenge, IAV-nanovax vaccinated mice showed similar levels of weight-loss but reduced respiratory distress (Penh) compared to naïve mice (Figures 5D,F). Furthermore, while 100% of naïve mice succumbed to the highly stringent IAV challenge, both prime only (40%) and prime+boost (80%) IAV-nanovax mice were protected from mortality (Figure 5E). Additionally, the protection mediated by IAV-nanovax appears durable as protection was still observed in mice challenged with homologous and heterologous virus at 100 days post vaccination (Figure 6). The ability to IAV-nanovax to confer protection against heterologous virus challenge is likely due to the local lung adaptive immune response induced by IAV-nanovax as IIV vaccinated mice, which lack lung Trm (Figure 4), had limited to no protection from a heterologous challenge (Supplemental Data Sheet 2D–F). Furthermore, this protection appears to require adaptive immunity specific to influenza as mice vaccinated with polyanhydride particles that only contained CpG and no IAV protein (CpG Particles) showed no pulmonary B or T cell responses (Supplemental Data Sheet 4A–D) and were not protected upon subsequent viral challenge (data not shown). Overall, these data suggest that IAV-nanovax induces a long-lived adaptive immune response that may confer significant protection against subsequent homologous and heterologous IAV exposures.

As previously described, we included a free antigen component in our vaccine as our prior results with s.c. vaccination had demonstrated that inclusion of this free antigen component enhanced immune responses and protection (25, 26). In order to determine if the free antigen component was likewise required during i. n. vaccination we next compared immune responses and protection in mice vaccinated with IAV-nanovax ± the free IAV antigens. As shown in Supplemental Data Sheet 4E–O, when the immune response in the lungs was examined at 32 days post vaccination lung-resident B cell numbers, GC B cell numbers, the fraction of class-switched GC B cells, the number of lung-resident antigen-experienced CD4 and CD8, as well as CD4 and CD8 Trm cells were equivalent or increased when the free antigen component was not administered as part of the vaccine. Likewise, IAV-specific IgG antibody titers in the serum were similar. Finally, no differences were observed in the ability of the vaccine to confer protection against a subsequent lethal dose homologous IAV challenge when IAV-nanovax vaccines were administered with or without a free antigen component were compared. Altogether these results suggest that a free antigen component is not required during i.n. IAV-nanovax vaccination to generate robust immunity and protection.

**Figure 7:**
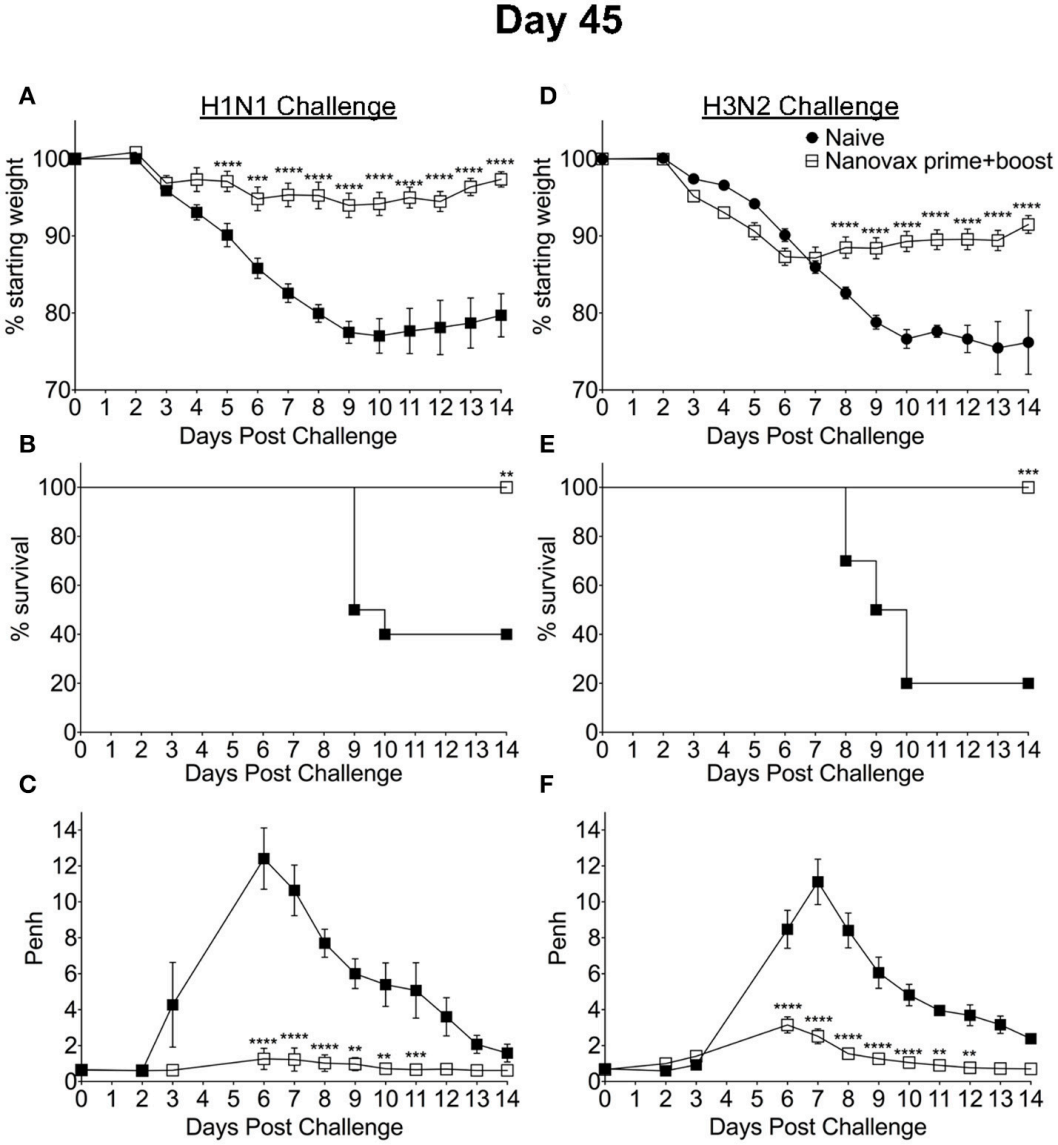
IAV-nanovax confers protection against subsequent homologous and heterologous IAV infection in outbred mice. Outbred Swiss Webster mice received a prime+boost i.n vaccination of IAV-nanovax without free protein or were left unvaccinated. Forty-five days following the initial vaccination, mice were challenged with a **(A–C)** 1108 TCIU dose of A/Puerto Rico/8/1934 (H1N1) or **(D–F)** 390 TCIU dose of A/Hong Kong/1/1968 (H3N2). Morbidity and mortality were measured by daily weight loss **(A,D)** and survival **(B,E). (C,F)** Penh was recorded daily as a measurement of lung function (airway resistance). Error bars, mean ± s.e.m. Data are representative of one independent with *n* = 10 mice/group. **(A,C,D,F)** ^**^P < 0.01, ^***^P < 0.001, ^****^P < 0.0001 (Two-way ANOVA with Holm-Sidak multiple-comparison test). **(B,E)** ^**^P < 0.01, ^***^P < 0.001 (Mantel-Cox Log rank test).

Our results presented have demonstrated the ability of IAV-nanovax to confer protection against IAV infections in an inbred C57BL/6 mouse model. The use of inbred models offers many advantages during the testing and design of vaccines, but these models do not represent the genetic diversity found in humans. Therefore, in order to determine if IAV-nanovax could likewise confer protection in outbred populations, we next i.n. vaccinated groups of outbred Swiss-Webster mice with IAV-nanovax. Groups of non-vaccinated mice were included as controls. These groups were then subsequently challenged on day 45 post vaccination with either lethal dose homologous (Figures 7A–C) or heterologous (Figures 7D–F) IAV. IAV-nanovax vaccination significantly reduced/ablated morbidity (weight loss, Figures 7A,D; Penh, Figures 7C,F) and protected from mortality (Figures 7B,E) upon subsequent challenges. Thus, these results demonstrate that IAV-nanovax is able to protect against subsequent homologous and heterologous IAV infections in a translational outbred model.

## Discussion

In the present study, we have demonstrated the efficacy of an i.n. administered CPTEG:CPH IAV-nanovax in producing IAV-specific immune responses and providing protection against subsequent homologous and heterologous IAV infections (Figures 5–7). While the protection provided by IAV-nanovax was found to be more robust after a prime+boost strategy, the prime only vaccination substantially reduced morbidity (Figures 5A,C) and completely prevented mortality (Figure 5B) following a homologous IAV challenge. Likewise, the prime only vaccination reduced initial airway distress (Figure 5F) and provided a significant level of protection from mortality during a lethal-dose heterologous IAV challenge (Figure 5E). This protection against homologous and heterologous virus appears to be long lasting as IAV-nanovax vaccination also conferred protection in mice challenged at 100 days post vaccination (Figure 6). While the IAV-nanovax formulation tested herein contained only IAV HA and NP proteins, studies have demonstrated that immunity directed against additional IAV proteins such as NA and M1 can enhance protection (47, 48). One of the benefits of our nanovaccine platform is the ability to easily “plug and play” new antigens within the formulation. Therefore, by incorporating IAV NA and M1 protein within future IAV-nanovax formulations we may be able to drive even more robust immunity and further improve protection against homologous and heterologous IAV infections.

Consistent with the ability to protect against homologous virus challenge, intranasal vaccination with IAV-nanovax induced IAV-specific class-switched GC B cell responses that were resident in the lung as well as robust local (IgG and IgA) and systemic (IgG) IAV-specific antibodies (Figures 2, 3). However, at some time points we did observe reduced IAV-specific IgG in IAV-nanovax vaccinated mice compared to IAV infection (day 45 serum, day 32 BAL, Figures 2B,D). This reduction could be related to additional antigens available for targeting following IAV infection since IAV-nanovax only contains IAV HA and NP proteins. Consistent with this idea, HAI titers in the serum and BAL following IAV-nanovax were similar to those observed for an IAV infection and well above the 1:40 HAI titer associated with protection (43) suggesting that HA-specific antibodies are equal.

Based on the observed protective ability of Trm against IAV, it has recently been suggested that a “universal” vaccine against IAV should induce such T cell responses in order to offer the greatest level of protection. Importantly, analysis of the lungs after IAV-nanovax vaccination found the presence of IAV-specific CD4 and CD8 T cells. These IAV-specific CD4 and CD8 T cells were within the lung parenchyma based on CD45 i.v.Ab exclusion staining (Figure 3) and expressed markers consistent with the canonical tissue-resident memory phenotypes. Lung-resident memory CD4 T cells are primarily identified by CD69 expression following infection or vaccination (45). While we observed CD69^pos^CD103^neg^ CD4 Trm subset within the lungs of IAV-nanovax vaccinated mice, we unexpectedly observed a small proportion of CD69^pos^CD103^pos^ CD4 T cells as well (Figures 4A,C). Although this CD4^pos^CD69^pos^CD103^pos^ resident memory phenotype has not been well characterized, a study has reported this subset within the skin (49). What role these CD69^pos^CD103^pos^ CD4 T cells may play in protection against subsequent IAV infections remains to be determined.

Previous studies have shown that the maintenance of Trm T cells within lung niches is influenced by the presence and longevity of antigen depots (18, 21, 23). Following IAV-nanovax vaccination, we observed the presence of both CD4 and CD8 Trm cells within the lungs on day 32 and 45 post vaccination at numbers similar to those observed in an IAV infected lung (Figure 4). Preliminary studies also suggest that CD4 and CD8 Trm responses are present in the lungs out to at least day 100 (data not shown). Our prior studies have shown nanoparticles persist within the lungs for ≥14 days and the continual release of antigen from nanoparticles placed into other tissues ≥30 days following vaccination. Overall this suggests that IAV-nanovax may act as an antigen depot, similar to what is observed during IAV infections (21, 22), and that this may contribute to the upkeep of lung-resident memory T cells.

In conclusion, we have shown that an i.n. inoculation with a polyanhydride nanovaccine encapsulating IAV proteins (IAV-nanovax) provides protection against homologous and heterologous IAV infections. This protection was associated with the induction of GC B cells in the lungs, robust IAV-specific antibody responses both systemically and locally, and IAV-specific CD4 and CD8 T cell responses within the lungs. Further, this report demonstrates for the first time that i.n. vaccination with polyanhydride nanoparticles can induce tissue-resident memory CD4 and CD8 T cells, confer protection against a heterologous virus challenge, and protect against infection in outbred populations. Altogether these findings highlight the potential of utilizing this nanovaccine platform for vaccine delivery in order to induce both systemic and localized adaptive immunity and provide protection against IAV infections.

## Author contributions

BN, TW, and KL conceived the research. ZZ, TW, and KL designed the experiments, analyzed data, and interpreted the results. ZZ conducted the experiments. EH provided technical assistance with processing of tissue samples and flow cytometry. KR, JG, and BN manufactured the IAV-nanovax. ZZ and KL wrote the manuscript and ZZ, KR, JG, TW, BN, and KL edited the manuscript. All authors reviewed and approved the manuscript.

### Conflict of interest statement

The authors declare that the research was conducted in the absence of any commercial or financial relationships that could be construed as a potential conflict of interest.
