# Supplementary material for: Polyanhydride Nanovaccine Induces Robust Pulmonary B and T Cell Immunity and Confers Protection Against Homologous and Heterologous Influenza A Virus Infections
Source: Front Immunol. 2018 Aug 28;9:1953. doi: 10.3389/fimmu.2018.01953 (PMC6127617; doi:10.3389/fimmu.2018.01953)
Supplement: Supplementary file 1 [file Data_Sheet_1.PDF]

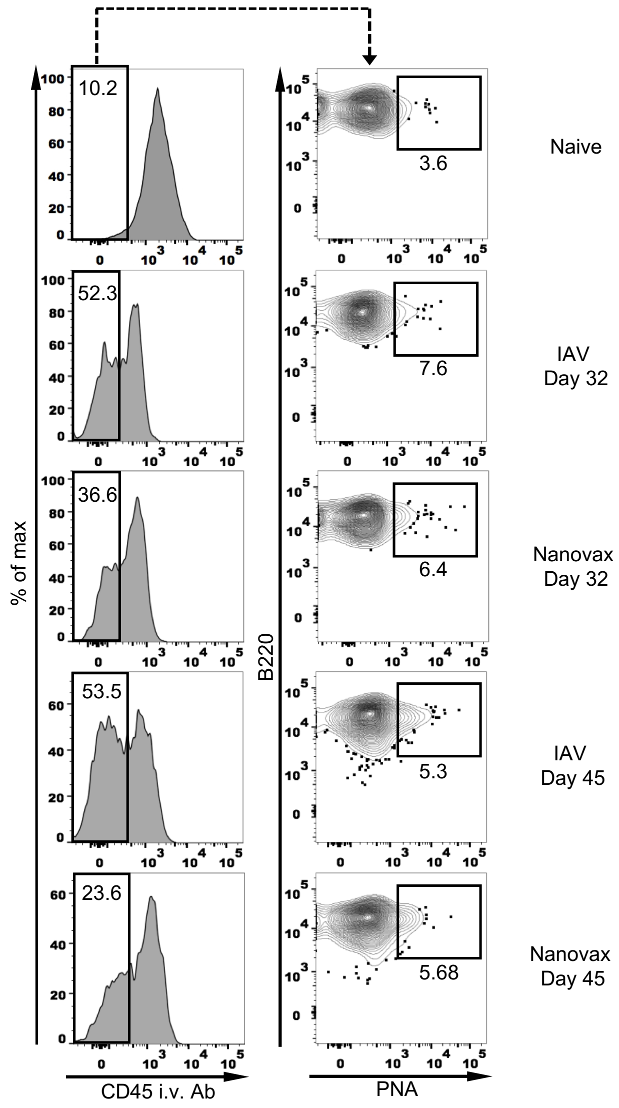

Supplemental Data Sheet 1. Lung resident GC B cell response gating strategy. C57BL/6 mice were challenged i.n. with 110 TCIU of A/Puerto Rico/8/1934, prime+boost vaccinated i.n. with IAV-nanovax or left unchallenged/ unvaccinated (naïve). At 32 and 45 days post challenge/vaccination, mice received fluorophore conjugated anti CD45.2 monoclonal antibody i.v. 3 minutes prior to harvesting the lungs. Lung resident B cells ($CD19^{+}B220^{+}$, left column) were gated as CD45 i.v.Ab^{neg}. Lung resident GC B cells $CD19^{+}B220^{+}CD45i.vAb^{neg}PNA^{pos}$ were determined by subsequent PNA gating (right column). Flow plots are representative of 3 independent (Day 32) or 1 independent (Day 45) experiments with n=4 mice/group.

Day 45

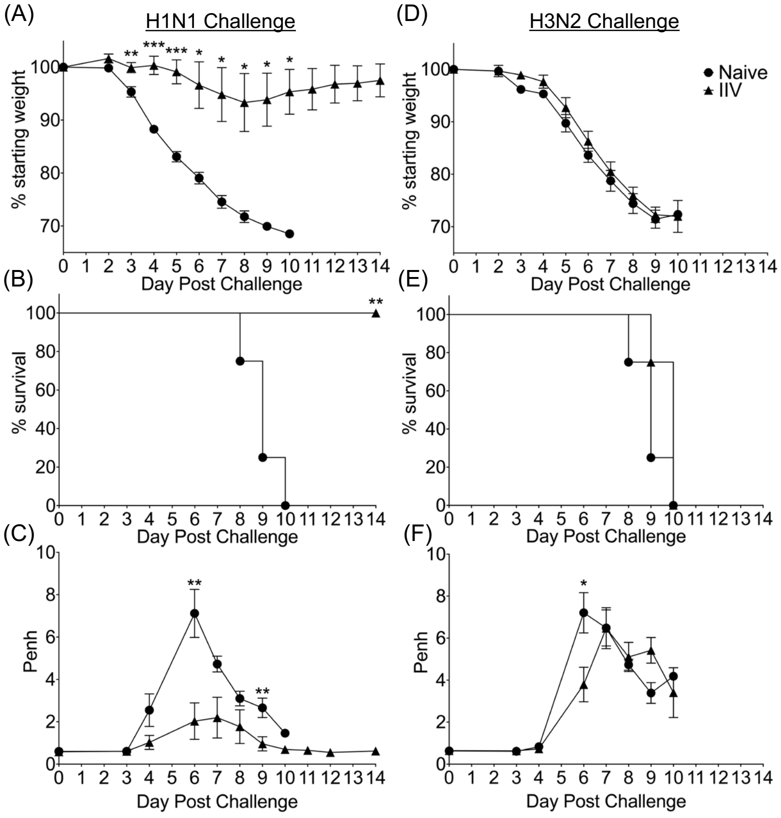

Supplemental Data Sheet 2. Intramuscular vaccination with IIV confers protection against subsequent homologous, but not heterologous, IAV infection. C57BL/6 mice received two-doses i.m. of IIV separated by 14 days or were left unvaccinated (naive). Forty-five days following the initial vaccination, mice were challenged with a (A-C) 1108 TCIU dose of A/Puerto Rico/8/1934 (H1N1) or (D-F) a 390 TCIU dose of A/Hong Kong/1/1968 (H3N2). Morbidity and mortality were measured by daily weight loss (A, D) and survival (B, E). (C, F) Penh was recorded daily as a measurement of lung function (airway resistance). Error bars, mean ± s.e.m. Data are representative of one independent experiment with n=5 mice/group. (A, C, D, E) *P<0.05, **P<0.01, ***P<0.001 (Two-way ANOVA with Holm-Sidak multiple-comparison test). (B, E) **P=0.01 (Mantel-Cox Log rank test).

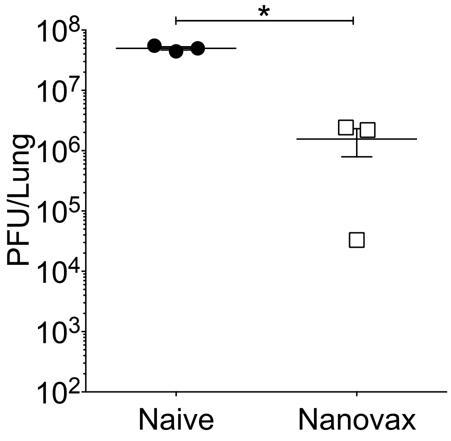

Supplemental Data Sheet 3. Vaccination with IAV-nanovax reduces viral titers within the lungs following challenge with homologous IAV. C57BL/6 mice received an i.n. prime+boost vaccination of IAV-nanovax or were left unvaccinated. 45 days after the initial vaccination they were challenged, as in Figure 1 with a 1108 TCIU dose of A/Puerto Rico/8/1934 (H1N1). At 3 days post infection, virus titers in lung homogenates were measured by plaque assays. **P*≤0.05 (two-tailed t test). Data are representative of 3 independent plaque assays with n=3 mice/group.

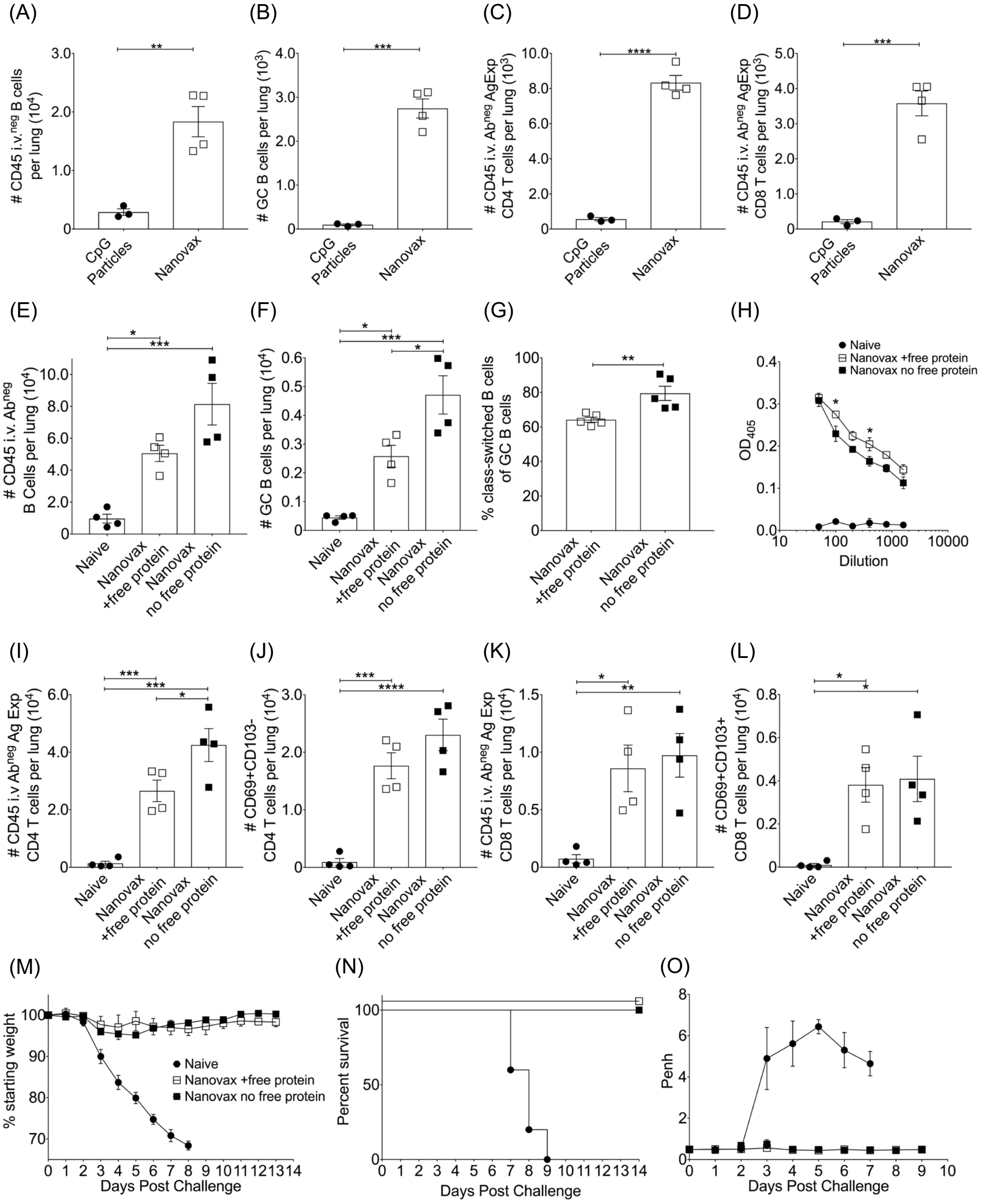

Supplemental Data Sheet 4. Immune response following vaccination with CpG only nanoparticles or IAV-nanovax without the free protein component. C57BL/6 mice received one dose i.n. of polyanhydride nanoparticles containing only CpG1668 (CpG Particles), two doses i.n. of IAV-nanovax (prime+boost) with or without free HA and NP protein, or were left unvaccinated (naive). At 32 (E-L) or 45 (A-D, M-O) days following the initial vaccination (A,E) lung-resident B cells, (B,F) germinal center (GC) B cells, (G) class switched B cells, (C,I) lung-resident CD4 T cells, (D, K) lung-resident CD8 T cells, and Trm cells (J,L) were enumerated within the lungs. Serum was also collected and (H) total IAV-specific IgG was quantified by ELISA. A group of mice were also challenged with a (M-O) 1108 TCIU dose of A/ Puerto Rico/8/1934 (H1N1). Morbidity and mortality were measured by daily weight loss (M) and survival (N). (O) Penh was recorded daily as a measurement of lung function (airway resistance). Error bars, mean ± s.e.m. Data are representative of one independent with n=4-5 mice/ group. *P<0.05,**P<0.01, ***P<0.001, ****P<0.0001 (A-D: Two-tailed student's t-test; E-G, I-L: One-way ANOVA with Tukey's multiple comparisons test; H, M, O: Two-way ANOVA with Holm-Sidak multiple comparisons test; N:Matel-Cox Log Rank Test).
